# PPARs: A Double-Edged Sword in Cancer Therapy?

**DOI:** 10.1155/2008/350351

**Published:** 2009-06-29

**Authors:** Dipak Panigrahy, Arja Kaipainen, Mark W. Kieran, Sui Huang

**Affiliations:** ^1^Vascular Biology Program, Children's Hospital Boston, Harvard Medical School, Boston, MA 02115, USA; ^2^Department of Pediatric Oncology, Dana-Farber Cancer Institute, Harvard Medical School, Boston, MA 02115, USA; ^3^Department of Biochemistry & Molecular Biology, University of Calgary, Calgary, AB, Canada T2N 4N1; ^4^Institute for Biocomplexity and Informatics, University of Calgary, Calgary, AB, Canada T2N 1N4

Welcome to this special issue of PPAR Research, PPARs: A Double-Edged Sword in Cancer Therapy. Peroxisome Proliferator-Activated Receptors (PPARs) are a family of pleiotropic transcription factors that play central roles in cell metabolism and regulation of inflammation. Cancer is thought to be the uncontrolled clonal evolution and expansion of a mutated cell [1]. What is the connection between PPARs and cancer? The theme of this special issue reflects the impressive confluence of two originally separate streams of investigation: PPARs and cancer research. Despite the multitude of points of intersection between PPARs and neoplasia, and associated unresolved paradoxes, the link between PPARs and tumors is not yet widely appreciated among specialists in either field. However, over the past years, investigators who cross these fields have unearthed a myriad of remarkable connections. 

In view of the multiple links between PPARs and cancer, perhaps epitomizing the pleiotropy of the biological effects of PPARs, this special issue contains an unusually large number of excellent contributions. This large volume may also reflect the increasing recognition of PPARs as a key player in cancer. To help guide the readers, we have organized the articles, in a departure from tradition, not according to the subtypes PPAR*α*, *δ*/*β* and *γ*, but instead, broadly in Sections 1–6. Section 1 contains reviews that offer a comprehensive overview on PPARs' effects in cancer and on the more established role of PPAR*γ* in cancer therapy. This is followed by Sections 2, 3, and 4 which have articles that discuss the following three key questions.

We close our special issue with Sections 5 and 6, which focus on PPAR ligand-based cancer therapies and the molecular mechanisms through which these ligands may act.

(1) We start with five reviews that provide the necessary background on structure and physiology of PPARs with an emphasis on their role in cancer. One of these reviews focuses on PPAR*γ*, which historically has been studied most intensely in conjunction with cancer. Notably, the therapeutic potential of PPAR*γ* agonists has been evaluated in clinical trials for liposarcoma and prostate cancer. In fact, 38 out of a total of 56 articles in this issue focus on PPAR*γ*.

(2) We continue with the first key question: *Do PPARs promote or inhibit cancer? * The opposite directions of observed PPAR effects on cancer—which gave this special issue its title—cannot be addressed in a straightforward manner. This is not because of ambiguous observations but (what makes it interesting) because the observed effects of PPAR on tumors have been clear—cut and powerful in either direction—either stimulating or suppressing tumors. That PPARs act as a “double-edged sword” may not come as a surprise to veterans of PPAR research who appreciate their pleiotropic effects. While PPAR*α* was the first PPAR to be associated with tumorigenesis, the emerging awareness of the PPAR*γ*-cancer connection is evidenced by the fact that 4 out of 7 reviews in this issue focus on PPAR*γ*.

(3) While much attention has been devoted to determining whether PPARs are friend or foe of tumors, our second question is also of fundamental significance*: Is PPAR's role in tumor growth mediated by cell autonomous or by noncell autonomous mechanisms?* From the perspective of PPAR investigators, this question may arise naturally because PPARs regulate intracellular processes, including proliferation, apoptosis, and differentiation as well as inflammatory processes through the control of mediators in cell-cell communication. In cancer biology this dualism has deeper roots. It is the subject of a major paradigm shift that has occurred over the past decade in cancer research. The simple notion, unquestioned for decades, that cancer is a cell-autonomous disease, driven by mutation and selection for fast growing and increasingly malignant cell clones, has yielded to the more encompassing view that cancer is also a nonautonomous disease, requiring the support from the “tissue microenvironment” in the “tumor bed”.

It took many years to overcome the picture of cancer cell autonomy afforded by cellular oncogenes. It began with a simple idea that had far-reaching consequences. Judah Folkman proposed in 1972, against all conventional wisdom, that tumor growth required neovascularization, and that such “tumor angiogenesis” was induced by soluble factors produced by the tumor. We dedicate this special issue to Dr. Folkman (1933–2008), our teacher and mentor, who has opened the world's eye to the tissue context of tumors. His arduous uphill battle against the established paradigm of cell-autonomous growth, although focused on angiogenesis, has shined the first beam of light on the role of the host microenvironment which was hidden in the shadow of the quest for mutations that establish the oncogenic pathways in the cancer cell. Dr. Folkman's persistence paved the path to the acceptance of the active role of nonneoplastic, “host” cells in the tumor microenvironment. In this generalization of the concept of tumor angiogenesis, it is now firmly established that the tumor stroma is comprised a variety of cells that are essential for tumor growth, including “tumor associated fibroblasts”, various inflammatory cells, and the pericytes around the tumor endothelium.

Much as cancer research was initially focused on the tumor parenchyma, the first connection between PPAR*α* and tumorigenesis was also directed at understanding how prolonged PPAR*α* activation by its ligands induces hepatocarcinogenesis in rodents by altering liver cell function [2]. However, mirroring the development in tumor biology, attention soon turned toward the effects of PPAR on the tumor microenvironment. In this issue, ten articles discuss the modulation of the tumor stroma by PPARs. Five of these reviews discuss their effects on the tumor endothelium, while the other five focus on the inflammatory compartment.

(4) The third major question addressed in this issue refers to the tumor-inducing or inhibiting effects of PPAR ligands: are their activities on tumors mediated by their nominal targets, the nuclear receptors, or do they act in a PPAR-independent manner? This matter is complicated by the fact that both PPAR agonists and antagonists can inhibit tumor progression. Six reviews provide an overview of the use of PPAR agonists and their “off-target” effects in various cancer therapies. We have also included one original research article on how rosiglitazone inhibits both tumor and endothelial cells via receptor dependent and independent mechanisms.

(5) The vast majority of PPAR research in the context of cancer focuses on the use of ligands in anticancer therapies. Thus, we dedicate the next section to articles that review preclinical and clinical studies of the use of PPAR*α* and PPAR*γ* ligands in a variety of cancer models, including combinatorial therapy.

(6) The last section of this special issue contains articles that review the molecular mechanisms through which PPARs, or their ligands, modulate tumor growth. There is an additional original research article in this section on how rosiglitazone inhibits tumor cell proliferation by interfering with IGF-IR signaling.

We hope you will find these articles informative. Clearly, much work lies ahead if we are to unravel the mysteries behind the double edged-sword nature of PPARs. This special issue describes the problem from many angles, and in doing so it reveals the gaps in our knowledge. Thus, rather than providing a unifying answer, it may hopefully inspire you to further research. 

*Dipak Panigrahy* *Dipak Panigrahy* *Arja Kapainen* *Arja Kapainen* *Mark W. Kieran* *Mark W. Kieran* *Sui Huang* *Sui Huang*
